# Assessment of heavy metals and naturally occurring radioactive materials (NORM) from historical mine wastelands: a case study of Brakpan, South Africa

**DOI:** 10.1007/s10661-026-15781-5

**Published:** 2026-08-21

**Authors:** Tobius Mawase Mogale, Memory Tekere, Khumbudzo Walter Maphangwa

**Affiliations:** Department of Environmental Sciences, University of South Africa, Science Campus, Johannesburg, 1710 South Africa

**Keywords:** Mine wastelands, Brakpan (Kwa-Thema South Africa), Heavy metals, NORMs, Environmental contamination, Radiological hazards

## Abstract

Mining has contributed to South Africa’s development, but its legacy has left more than 6000 abandoned sites, with Brakpan in South Africa among the most affected areas. This study assessed heavy metals and naturally occurring radioactive materials (NORM) in the Brakpan mine wasteland in Gauteng Province, South Africa. Soil and water samples from the area were analysed using inductively coupled plasma mass spectrometry (ICP-MS) for heavy metals and high-purity germanium gamma-ray spectrometry (HPGe) was used for NORM analysis. The results showed elevated concentrations of several contaminants. In soils, chromium (103.98 mg/kg) and arsenic (47.75 mg/kg) were notably enriched, while uranium exhibited the highest mean concentration (7.14 mg/kg), exceeding the South African soil quality guidelines for contaminated land. Water samples showed high concentration of uranium (mean 1.64 mg/L) and nickel (12.78 mg/L) concentrations, exceeding guideline values for agricultural use and aquatic ecosystem protection value of 0.03 mg/L and 0.04 mg/L, respectively. Suggesting potential risks to downstream water users and ecological receptors. Radiological analysis revealed activity concentrations of ^238^U ranging from 12.78 to 409.73 Bq/kg (mean: 160 Bq/kg), with mean activity concentrations of ^232^Th and ^40^ K of 19.65 Bq/kg and 376 Bq/kg, respectively. The absorbed dose rate averaged 108.19 nGy/h exceeding the UNSCEAR global average background level (59 nGy/h). Localised hotspots exhibited radium equivalent activity of up to 492.49 Bq/kg and an external hazard index of 1.33, indicating elevated radiological risk near residential settlements. Overall, the findings highlight combined geochemical and radiological hazards, underscoring the need for integrated remediation and management strategies to protect affected communities.

## Introduction

Historical mining has left a widespread legacy of environmental contamination in many regions worldwide, particularly in areas where gold, uranium, copper and polymetallic ores were extracted over long periods. In Europe, abandoned sulphide mines in the Iberian Pyrite Belt (Spain and Portugal) continue to release arsenic, copper, lead and zinc into surrounding soils and river systems decades after closure (Martín-Méndez et al., 2023; Mourinha et al., 2022; Santos et al., 2024). Similar contamination has been reported in Asia, where gold and rare metal mining in China has resulted in the mobilisation of chromium, copper and arsenic through tailings and acid mine drainage (AMD) (Zhao et al., 2022). In North America, former uranium-mining regions of the Colorado Plateau and parts of Canada continue to exhibit elevated uranium and radium concentrations in soil and groundwater systems long after mine closure (Ingram et al., 2017; Proulx et al., 2025; USEPA, 2025) . These cases demonstrate that heavy metals and naturally occurring radioactive materials (NORM) remain long-term environmental hazards where historical rehabilitation was inadequate.

The environmental and health impacts associated with mine wasteland contamination are well documented across regions of the world (IAEA, 2023; CER, 2025; USEPA, 2025). Exposure to metals such as uranium, arsenic, cadmium, chromium and copper through drinking water, irrigation and food chain transfer has been linked to respiratory disorder, renal dysfunction, neurological effects and an increased cancer risk in affected populations (WHO, 2017; 2022; Gibb et al., 2021; Dehkordi et al., 2024). In uranium-rich environments, enhanced gamma radiation from tailings contributes to external exposure risks for nearby communities (UNSCEAR, 2021; IAEA, 2023). These impacts are most severe where abandoned mine residues overlap with settlements, agricultural land and water resources, a pattern observed globally in both developed and developing mining economies (Kazapoe et al., 2023; Wolkersdorfer et al., 2021).

Across Africa, legacy of historical mining continues to challenge environmental management systems (EMS). In Ghana, abandoned gold mining areas such as Obuasi have contaminated rivers and soils with arsenic and mercury, posing long-term risks to food safety and drinking water quality (Kazapoe et al., 2023). Similarly, in Zambia’s Copperbelt, high concentrations of copper and cobalt persist near historical tailings and smelters (Dusengemungu et al., 2022; Kríbek et al., 2023). In Namibia, uranium mining in the Erongo region has resulted in elevated natural radioactivity in soils and groundwater systems (Abiye & Shaduka, 2017; Indongo & Mathuthu, 2023). Despite these impacts, many African mine wastelands remain unmanaged due to financial, institutional and regulatory constraints.

In South Africa, mining has played a central role in national economic development, and this legacy is associated with more than 6000 abandoned and ownerless mines that now represent significant environmental and socioeconomic risks (CER, 2025; Manyane, 2025). Gold mining within the Witwatersrand Basin has generated extensive tailings material enriched with heavy metals and NORMs, much of which predates modern environmental legislation. These tailings remain major sources of acid mine drainage (AMD), dust emissions and groundwater contamination (Dusengemungu et al., 2022; Naicker et al., 2021). Elevated concentrations of uranium, arsenic and chromium have been widely reported in Gauteng, Limpopo and Free State provinces (Hobbs et al., 2022; Naicker et al., 2021).

Brakpan, located in the Eastern Witwatersrand of Gauteng province, South Africa, represents one of the most affected areas, with extensive mine wastelands adjacent to densely populated communities such as Kwa-Thema, Langaville and Dunnottar. These disturbed landscapes contain tailings and contaminated soils enriched with heavy metals and NORMs (Mohuba et al., 2025; DMRE, SA. 2023/2024; Zupunski et al., 2023). The close proximity to residential areas, surface water systems and informal land use makes Brakpan a particularly sensitive and high-risk environment, further exacerbated by the absence of effective rehabilitation measures.

While international experience from countries such as Australia and Canada demonstrates that abandoned mine sites can be stabilised through structured scientific assessments, monitoring and integrated remediation strategies (Bussière et al., 2022; Jones et al., 2020), there remains a critical need within South Africa for site-specific scientific data on the distribution, behaviour and associated risks of heavy metals and NORMs to effectively inform remediation planning, policy development and long-term land use management (Mogashane et al., 2025; Mohuba et al., 2025).

This study evaluated the levels of heavy metals and NORMs in the Brakpan mine wasteland. The study includes quantification of contamination levels in soil and water, the identification of potential hotspots and the assessment of environmental and radiological risks the results of which provide evidence to support informed remediation and management strategies. By placing Brakpan within both the African and global context of mine wasteland contamination, this study contributes to ongoing efforts aimed at sustainable mine closure, environmental rehabilitation and the protection of vulnerable communities within the Witwatersrand Basin and comparable mining regions internationally.

## Material and methods

### Area of study

**Fig. 1:**
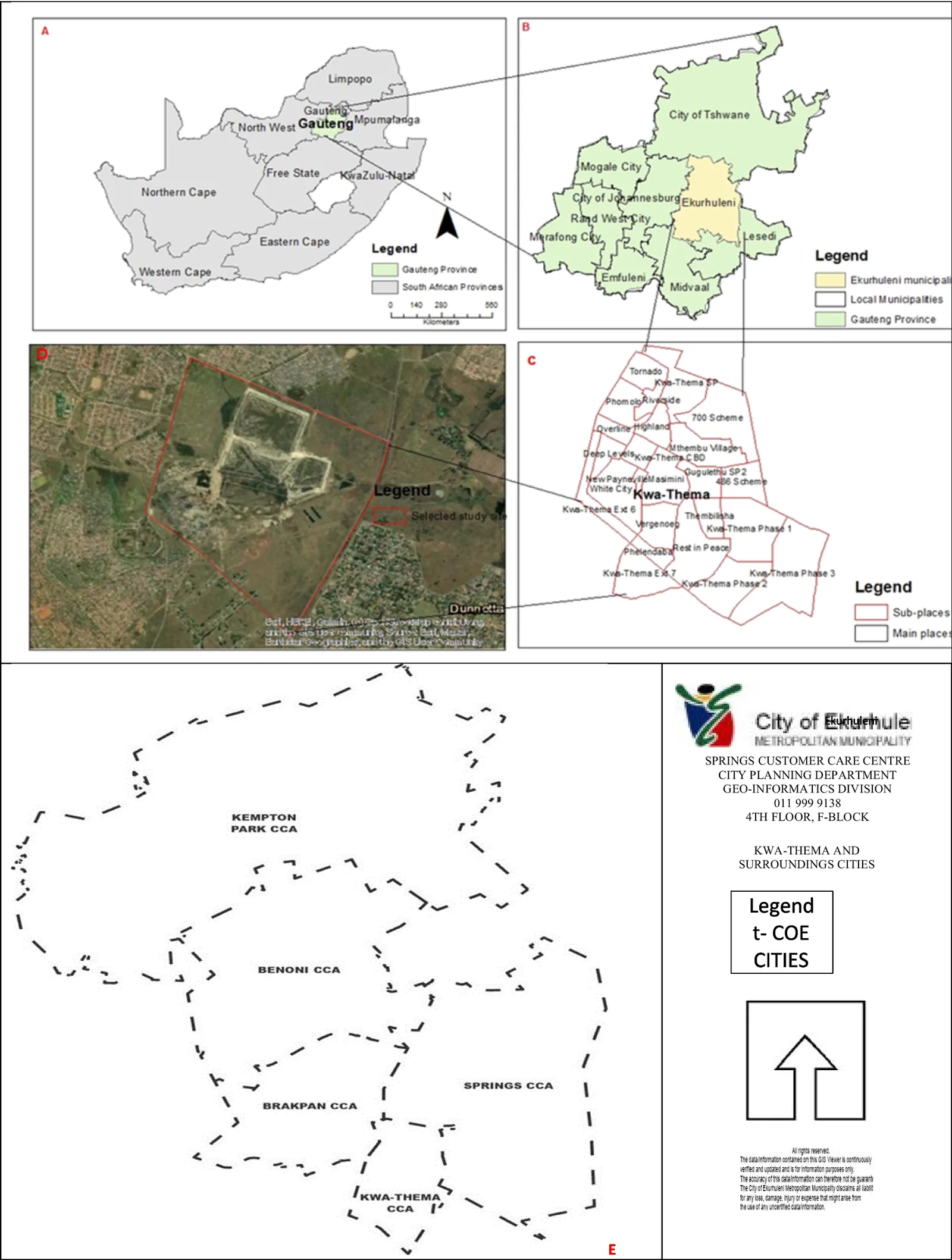
Brakpan map (mine dump)

This study was conducted in Brakpan, a town within the Ekurhuleni Metropolitan Municipality (EMM) in Gauteng Province, South Africa. The specific sampling locations are situated within Kwa-Thema, a township that forms part of the greater Brakpan area within EMM. Brakpan is situated at 26°19ʹ41.67ʺ S and 28°25ʹ02.41ʺ E, at an elevation of approximately 1616 m (5300 ft) above sea level. The town lies between Springs and Benoni and to the south of Kempton Park, within the eastern part of Gauteng Province (Fig. 1). Brakpan has a temperate climate characterised by warm, wet summers and dry winters. The total land area is 182.81 km^2^ (70.58 sq. mi), with a diverse population of approximately 305,692 residents, including the subarea Kwa-Thema with 103,727 people (Stats-SA, 2018; WPR, 2025). Geographically, the study area lies within the Witwatersrand Basin, characterised by gold- and uranium-bearing sedimentary formations. Geological structures such as faults and fractures influence groundwater flow and facilitate the mobilisation of heavy metals and radionuclides from mine tailings into surrounding soils and surface water systems (Mohuba et al., 2025; Naicker et al., 2021). The area has historically been associated with uranium and gold mining following discoveries of gold and coal between year 1888 and 1905. The biome is grassland, typical of the Highveld, with soils ranging from clay to sand, often altered by mining and urban development (Prinsloo et al., 2021). Brakpan is unique in that it accommodates a range of industries, particularly mining, construction and retail. Although the town lacks major landmarks or monuments, increasing infrastructure development and economic activities have significantly transformed land use. Mining remains a central economic activity, while sectors such as retail, transportation and tourism also play vital roles in supporting the gross domestic product (GDP) of the Ekurhuleni region (Stats-SA, 2018; CoE, 2023).

### Sampling design

Sampling was conducted once per site in November 2024 to assess the spatial patterns of heavy metals and NORMs contamination in soil and surface water within the mine wasteland. A GIS-based grid was applied to the study area to ensure adequate spatial coverage of the sampling sites. The grid resolution was defined based on site accessibility and the spatial extent of the study area to ensure representative coverage of heterogeneous zones. Sampling points were selected using a combination of random and purposive sampling approaches. Random sampling was used to capture background spatial variability across the study area, while purposive sampling targeted visibly disturbed zones, including tailings deposition areas, surface runoff pathways and zones of direct human and livestock activity where contaminant mobilisation was considered most likely. In total, 16 soil samples and 5 surface water samples were collected from the demarcated sites. This sampling density was sufficient to capture spatial variability across the study area, including both background conditions and contamination hotspots. Sampling was conducted during a single season and therefore represents conditions at the time of sampling; however, the results are considered sufficient for assessing spatial patterns of contamination within the study area. Background control samples were collected from Mamelodi East, a site located approximately 50 km from the study area, selected based on the absence of mining activities and relatively stable environmental conditions.

### Sample collection

#### Soil

**Fig. 2:**
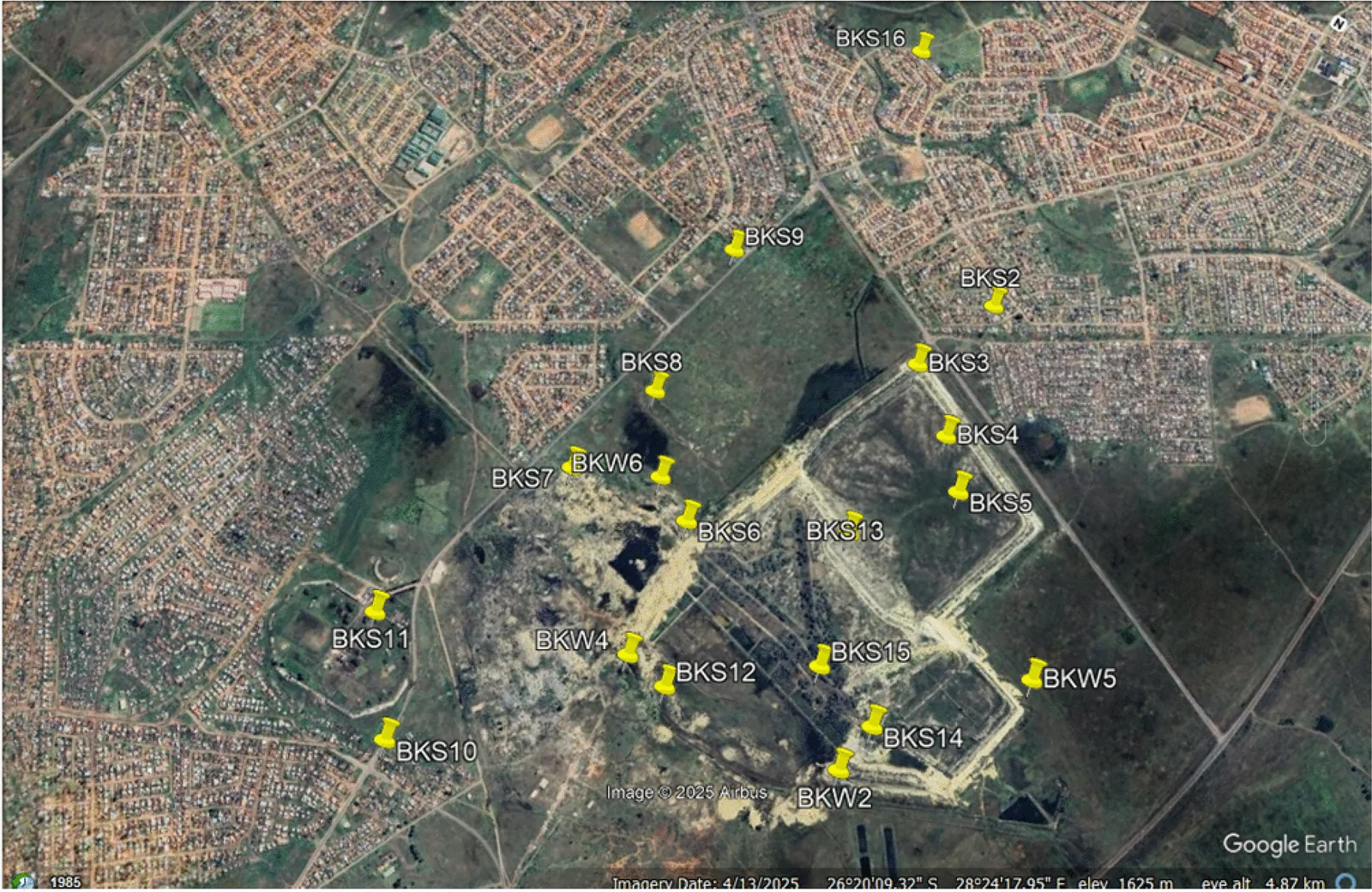
Google map image of Brakpan (Kwa-Thema) where the samples were collected

A total of 16 soil samples were collected using spades and inert amber glass containers (l liter), with the top 15 cm soil was removed to avoid anthropogenic surface layers that might distort the results of both heavy metal concentrations and radionuclide activities (Islam et al., 2024). Approximately 500 g to 1 kg of soil sample was collected from each site, and the coordinates of each point were recorded using a global positioning system (GPS) as shown in Fig. 2 and Table 1. Samples were labelled according to their location (e.g., BKS for Brakpan soil sample and transported to the laboratory for analysis). Subsamples were selected for ICP-MS analysis, and all 16 samples were analysed using a high-purity germanium (HPGe) detector for gamma-ray spectrometry. Standardised environmental sampling procedures were followed throughout.

**Table 1:** The GPS coordinates for collected soil and water samples from mine wasteland BP and BKS are sites for soil samples, BPW and BKW are sites for water samples and BPW for control sample)

Sampling point	Location		Description of sampling points
	Latitude	Longitude	
BP1 (control sample)	−25.735206	28.455628	Located at Mamelodi East about 50 km away from study area
BKS2	−26.323781	28.416044	Located near the tip of the mine wasteland, Kwa-Thema phase 2
BKS3	−26.328019	28.416531	
BKS4	−26.323783	28.416028	
BKS5	−26.328367	28.416058	
BKS6	−26.277425	28.428542	Located near tip of the mine wasteland, Langaville township (Masibeni street)
BKS7	−26.322156	28.410847	
BKS8	−26.307625	28.391417	
BKS9	−26.326011	28.40565	
BKS10	−26.420461	28.531828	Located near tip back corner of the mine wasteland near Langaville township
BKS11	−26.314772	28.4719	
BKS12	−26.336494	28.428542	
BKS13	−26.331033	28.407931	Located at the middle of the mine wasteland
BKS14	−26.334769	28.399094	Located at the front site of the mine wasteland (Tonk Meter Rd)
BKS15	−26.334442	28.411147	
BKS16	−26.326139	28.440589	Located at tip point away from mine wasteland (Kwa-Thema phase 2)
Water samples			
BPW1 (control sample)	−25.735219	28.455642	Located at Mamelodi East about 50 km away from the study area
BKW2	−26.336828	28.409414	Located at the back of mine wasteland (Longville and Dunnottar communities)
BKW3	−26.336411	28.408569	
BKW4	−26.331906	28.417842	Located at the front site of the mine wasteland (Tonk Meter Rd)
BKW5	−26.332628	28.406842	
Soil samples			
BKS11	−26.314772	28.4719	Located near tip back corner of the mine wasteland near Langaville township
BKS9	−26.326011	28.40565	Located near tip of the mine wasteland, Langaville township (Masibeni street)
BKS15	−26.334442	28.411147	Located at the front site of the mine wasteland (Tonk Meter Rd)
BKS16	−26.326139	28.440589	Located at tip point away from mine wasteland (Kwa-Thema phase 2)

#### Water

Surface water samples were collected from open surface water bodies (including streams, runoff channels and stagnant pools associated with AMD) using acid-cleaned 330 mL amber glass bottles. Each container was rinsed twice with water from the site before sampling and then dipped approximately 5 cm below the surface to collect about 150 mL of water. Then, 2 mL of ultrapure concentrated nitric acid (NHO_3_, 65%) was added immediately to preserve radionuclides and heavy metals. Samples were labelled with the code BKW (Brakpan water) and transported to the laboratory for analysis. GPS coordinates were recorded for all sampling points as shown in Fig. 2 and Table 1.

Both soil and water sampling followed standardised environmental protocols (International Standard Organisation (ISO) 11466: 1995 and ISO 5667-3:2018 guidelines (ISO, 2024)), to ensure accuracy, reliability and comparability of results. Control samples (BP1 and BPW1) were collected from a nonmining area in Mamelodi East (GPS coordinates, −25.735206 and 28.455628), approximately 50 km from the study area, to establish background levels.

### Sample preparation and extraction

After field collection, soil samples were air-dried, sieved to < 2 mm and homogenised. One gram of each soil sample was digested in a closed microwave vessel using aqua regia (HCl: HNO₃, 3:1; 9.0 mL HCl + 3.0 mL HNO₃). The mixtures were heated in a microwave, over 15 min and held for 20 min. After cooling, digestates were transferred into 50-mL polypropylene tubes, filtered through 0.45-μm membranes where necessary, and diluted to 50.0 mL with deionised water. The applied aqua regia digestion represents a pseudo-total extraction, as it does not fully dissolve silicate-bound elements; therefore, reported concentrations reflect the environmentally available fraction rather than absolute total concentration. Filtered water samples were acidified and digested under similar conditions and diluted to fixed volumes. Aliquots of the final solutions were spiked with internal standards and analysed by ICP-MS. Method blanks, laboratory control samples and certified reference materials were included with each batch; recoveries and detection limits were determined and used to validate results.

For radionuclide analysis, each soil sample was air-dried for 2 weeks to ensure the samples were free of moisture and then sieved with < 2 mm to obtain a homogeneous particle size. Dried soil samples were sealed in Marinelli beaker using masking tape for 26 days to allow secular equilibrium between parent radionuclides and their decay products before being analysed using gamma-ray spectrometry. These steps represent the full process, from sampling to instrumental measurement. The use of these experimental methods ensured reliable and valid environmental measurements and supported the assessment of contamination associated with the mine wasteland. Such laboratory-based evaluations of soil and water contamination are widely recognised in environmental research (Belkouteb et al., 2024). All analyses were performed following established quality assurance and quality control (QA/QC) procedures, including method blanks, calibration verification and the use of reference materials, to ensure the accuracy and consistency of the results (USEPA, 2022).

### Analysis of the heavy metals and NORMs

#### ICP-MS analysis

Heavy metals were quantified using a PerkinElmer NexION 2000 C ICP-MS in TotalQuant mode (PerkinElmer, 2023). Calibration was performed using certified multielement standards, with blanks, calibration checks and duplicates included to maintain analytical quality. Helium-based kinetic energy discrimination was used to minimise spectral interferences for metals including arsenic (As), lead (Pb), cadmium (Cd), chromium (Cr), copper (Cu), nickel (Ni), zinc (Zn), cobalt (Co) and uranium (U) (USEPA, 2022; Belkouteb et al., 2024). Concentrations were calculated on a dry-weight basis:

1 $$Concentration\;(dryweight)\;(\mu g/g)=\frac{C\cdot V_f\cdot DF}{W\cdot S\cdot1000}$$

where C is instrument reading (µg/L), ${V}_{f}$ final volume (mL), DF dilution factor, W dry sample mass (g) and S solids fraction.

Limits of detection (LOD) and limits of quantification (LOQ) were determined from instrument sensitivity and blanks. LOD values ranged from < 0.002 mg/kg (soil) to < 0.00002– < 0.00060 mg/L (water), with LOQ defined as three times the LOD. Recoveries (85–115%) confirmed analytical accuracy. Calibration used multielement standards, with uncertainty within ± 5–10%. TotalQuant mode supported multielement screening, and the solids fraction (S) was applied in dry-weight calculations.

#### Gamma-ray spectrometry analysis

A gamma-ray spectrometry system was used to determine the activity concentrations of radionuclides in the soil samples. The detector employed was an HPGe GCD-35–190, with a relative efficiency of 36% compared with a 3″ × 3″ NaI (Tl) detector and a full-width at half-maximum (FWHM) of 0.850 keV at the 122 keV photon energy of Co-57. The detector was housed in a graded shield consisting of lead (2.5 cm) and copper (12.0 cm) layers to attenuate external background radiation during counting. One of the main advantages of HPGe detectors is their high energy resolution, supported by a multichannel analyser (MCA) with 8192 channels, an analog-to-digital converter, memory, control logic and display (Knoll, 2020; IAEA, 2022).

Each soil sample was placed over the detector and counted for a minimum of 12 h to obtain high-resolution gamma spectra. System operation, spectral acquisition and data analysis were performed using the Genie 2000 software. Background spectra recorded before and after sample counting were subtracted from the sample spectra to ensure accuracy. For efficiency calibration, three reference powdered standards (RGU-1, RGTh-1 and RGK-1) obtained from the International Atomic Energy Agency (IAEA, 1987) were used for U, K and Th activity measurements.

The minimum detectable activity (MDA) for gamma-ray spectrometry was determined based on counting statistics, background radiation and detector efficiency. The MDA values were approximately 2–5 Bq/kg for ^238^U and ^232^Th and 10–20 Bq/kg for ^40^ K, based on a counting time of 12 h and sample-specific characteristics. Activity concentrations were determined using characteristic photopeaks for each radionuclide. For ^238^U, activity was derived from its progeny ^234^Th (63.200 keV and 92.600 keV) and ^234 mPa^ (63.200 keV). ^226^Ra activity was calculated from the gamma lines of its daughter nuclides: ^214^Pb (351.932 keV and 295.224 keV) and ^214^Bi (609.312 keV, 1120.287 keV and 1764.494 keV). For ^232^Th, the photopeaks of ^228^Ac (969.000 keV and 911.204 keV) and ^212^Bi (727.330 keV) were used. The activity concentration of ^40^ K was derived from its 1460.630 keV photopeak.

Activity concentrations were calculated using the standard gamma-spectrometric equation, assuming secular equilibrium in the ^238^U decay series:

2 $$A=\frac{{C}_{n}}{\varepsilon {P}_{\gamma }tm}$$

where A is the activity concentration (Bq/kg or Bq/L), ${C}_{n}$ net count rate, ε detector efficiency, ${P}_{\gamma }$ gamma emission probability, t counting time and m sample mass (kg).

### Determination of activity concentrations and radiological indices

Numerous radiological hazard indices are used to represent the radiation levels and their radiological impacts in terms of radiation dose assessments and dosimetric estimations. These radiological parameters used in this present study include absorbed dose rate (*D*), radium equivalent activity (Ra_eq_), external radiation hazard index (*H*ₑ*x*) and representative level index (*I*_ɣr_) which were determined from measured data. These parameters have been established based on equations reported in the literature and have been recognised by NNR, IAEA, ICRP and other agencies, demonstrating the reliability of these equations (NNR, 2021; IAEA, 2023).

#### Absorbed dose rate (D)

The absorbed dose rate (*D*) was calculated to estimate the external gamma radiation emitted from soil, based on established conversion factors used in global radiation protection literature. This index links the measured activities of ^238^U, ^232^Th and ^4^⁰K to the energy deposited in the environment (Kurnaz et al., 2020; UNSCEAR, 2021). The equation (Eq. 3) shown below was used.

3 $$D=0.462A_u+0.604A_{Th}+0.0417A_K$$

where *A*_*U*_, *A*_*Th*_ and *A*_*K*_ are activity concentrations of ^238^U, ^232^Th and ^4^⁰K in Bqkg^−1^and 0.462, 0.604 and 0.0417 the corresponding standard dose conversion factors.

#### Radium equivalent activity (Ra_eq_)

Radium equivalent activity (Ra_eq_) was calculated to integrate the combined gamma output of the three radionuclides into a single hazard index that allows direct comparison with widely used radiation safety criteria (Sowole & Egunjobi, 2019). This parameter remains one of the most common indicators in environmental radioactivity studies and Eq. (4) was employed.

4 $$Ra_{eq}=A_U+1.43A_{Th}+0.077A_K$$

where *A*_*U*_, *A*_*Th*_ and *A*_*K*_ are the specific activity concentration of ^238^U, ^232^Th and ^40^K in Bq/kg, respectively, and this formula is based on the calculation that 1 Bq/kg of ^238^U, 0.7 Bq/kg of ^232^Th and 13.0 of ^40^ K emit same gamma dose rate (Joel et al., 2021).

#### External hazard index (Hₑx)

The external hazard index (*H*ₑ*x*) was calculated to evaluate compliance with international exposure limits, based on the assumption that *H*ₑ*x* values not exceeding unity indicate acceptable radiation levels (Mustapha et al., 2024). The *H*ₑ*x* can be calculated from Eq. (5).

5 $$H_ex=\frac{A_U}{370}+\frac{A_{Th}}{259}+\frac{A_K}{4810}\leq1$$

whereby *A*_*U*_, *A*_*Th*_ and *A*_*K*_ are the specific activity concentration of ^23^⁸U, ^232^Th and ^40^K in Bq/kg, respectively.

#### Representative level index (Iγr)

The representative level index (*I*_γr_) was calculated as an additional screening tool, particularly useful for identifying materials that may pose elevated gamma-radiation risks under normal environmental conditions (Nair & Udupa, 2024). The index can be estimated using the following equation:

6 $$I_{yr}=\frac{A_U}{150}+\frac{A_{Th}}{100}+\frac{A_K}{1500}$$

where A_*U*_, A_*Th*_ and A_*K*_ are the specific activity concentrations of ^22^⁶Ra (^23^⁸U), ^232^Th and ^4^⁰K in Bq/kg. A representative level index of *I*_γr_ ≤ 2 corresponds to 0.3 mSv/year, while *I*_γr_ ≤ 6 corresponds to 1 mSv/year, according to the European Commission (1999).

These indices provide a robust framework for evaluating potential radiological hazards.

### Quality assurance and data processing

All analyses followed ISO/IEC 17025 standards, including calibration verification, sample geometry control and the use of certified reference materials. Gamma-ray spectrometry incorporated routine background monitoring and radiation control, with spectra processed using standard software, while ICP-MS data were processed using Syngistix (ISO, 2018; IAEA, 2022). Detection limits, recovery efficiencies and measurement uncertainty were quantitatively evaluated and found to fall within acceptable analytical ranges, supporting the reliability of the results. This ensured traceable and reproducible results aligned with international standards.

### Heavy metal concentrations

The concentrations of heavy metals detected in soil and water samples collected from the mine wasteland provide an overview of the spatial distribution and magnitude of contamination across the sampled locations and form the basis for interpreting potential environmental and human-health risks. Table 2 provides an overview of the concentrations of the major metals identified in the soil and water matrices.

**Table 2:** Measured concentrations of heavy metals in soil and surface water samples

Soil samples							
Heavy metals	BP1 (mg/kg)	BKS11 (mg/kg)	BKS9 (mg/kg)	BKS15 (mg/kg)	BKS16 (mg/kg)	Average (mg/kg)	Regulatory limits (mg/kg)
Pb	9.125	16.885	7.742	11.368	7.635	10.55 ± 3.85	20
Cu	5.009	26.737	47.193	33.966	51.057	32.79 ± 18.38	16
Cr	31.654	109.822	122.190	156.802	99.446	103.98 ± 45.85	6.5
Ni	9.799	58.083	78.462	129.081	78.618	70.81 ± 43.01	91
Cd	< 0.002	0.012	0.005	0.021	< 0.002	0.01 ± 0.01	7.5
Co	1.806	8.934	18.493	27.840	11.986	13.81 ± 9.87	300
Zn	17.678	34.720	17.658	46.311	19.029	27.08 ± 12.94	240
As	1.005	58.299	71.102	56.128	52.223	47.75 ± 27.07	5.8
V	10.630	18.161	17.270	25.280	16.155	17.50 ± 5.24	150
U	0.367	7.765	10.731	7.665	9.160	7.14 ± 3.99	2.29
Water samples							
Heavy metals	BPW1 (mg/L)	BKW2 (mg/L)	BKW3 (mg/L)	BKW4 (mg/L)	BKW5 (mg/L)	Average (mg/L)	Regulatory limits (mg/L)
Pb	< 0.00004	< 0.00004	< 0.00004	< 0.00004	< 0.00004		0.200
Cu	< 0.00019	< 0.00019	1.033	1.293	2.265	1.530 ± 0.65	0.200
Cr	< 0.00060	< 0.00060	< 0.00060	< 0.00060	< 0.00060		0.100
Ni	< 0.00012	< 0.00012	12.416	9.127	16.799	12.781 ± 3.85	0.200
Cd	< 0.00008	< 0.00008	0.004	0.003	0.005	0.004 ± 0.001	0.010
Co	< 0.00002	0.00022	3.811	2.812	5.142	2.941 ± 2.18	
Zn	< 0.00029	< 0.00029	3.434	2.424	4.423	3.437 ± 1.00	2.000
As	< 0.00006	0.00618	0.0081	0.0105	0.015	0.010 ± 0.004	0.100
V	< 0.00050	< 0.00050	< 0.00050	< 0.00050	< 0.00050		
U	< 0.00006	< 0.00006	1.1634	1.144	2.625	1.644 ± 0.85	0.030

Values reported as < 0.01 mg/kg suggest that the concentrations were too low to be detected by the instrument (PerkinElmer, 2023)

Table 2 shows that metals vanadium (V), chromium (Cr), cobalt (Co), nickel (Ni), copper (Cu), zinc (Zn), arsenic (As), cadmium (Cd), lead (Pb) and uranium (U) were detected at varying levels in soil samples collected from the Brakpan mine wasteland (BKS9, BKS11, BKS15 and BKS16) and in Brakpan water samples (BKW2, BKW3, BKW4 and BKW5), with control samples represented by BP1 and BPW1. Heavy metal concentrations in soil ranged from below detection limits (< 0.002 mg/kg) for Cd to 156.802 mg/kg for Cr, indicating substantial variability across the mine wasteland. In water samples, concentrations ranged from below detection limit for Cd to 16.799 mg/L for Ni. These values reflect elevated contamination in several locations and highlight areas where metals exceed typical background levels established from control samples.

**Fig. 3:**
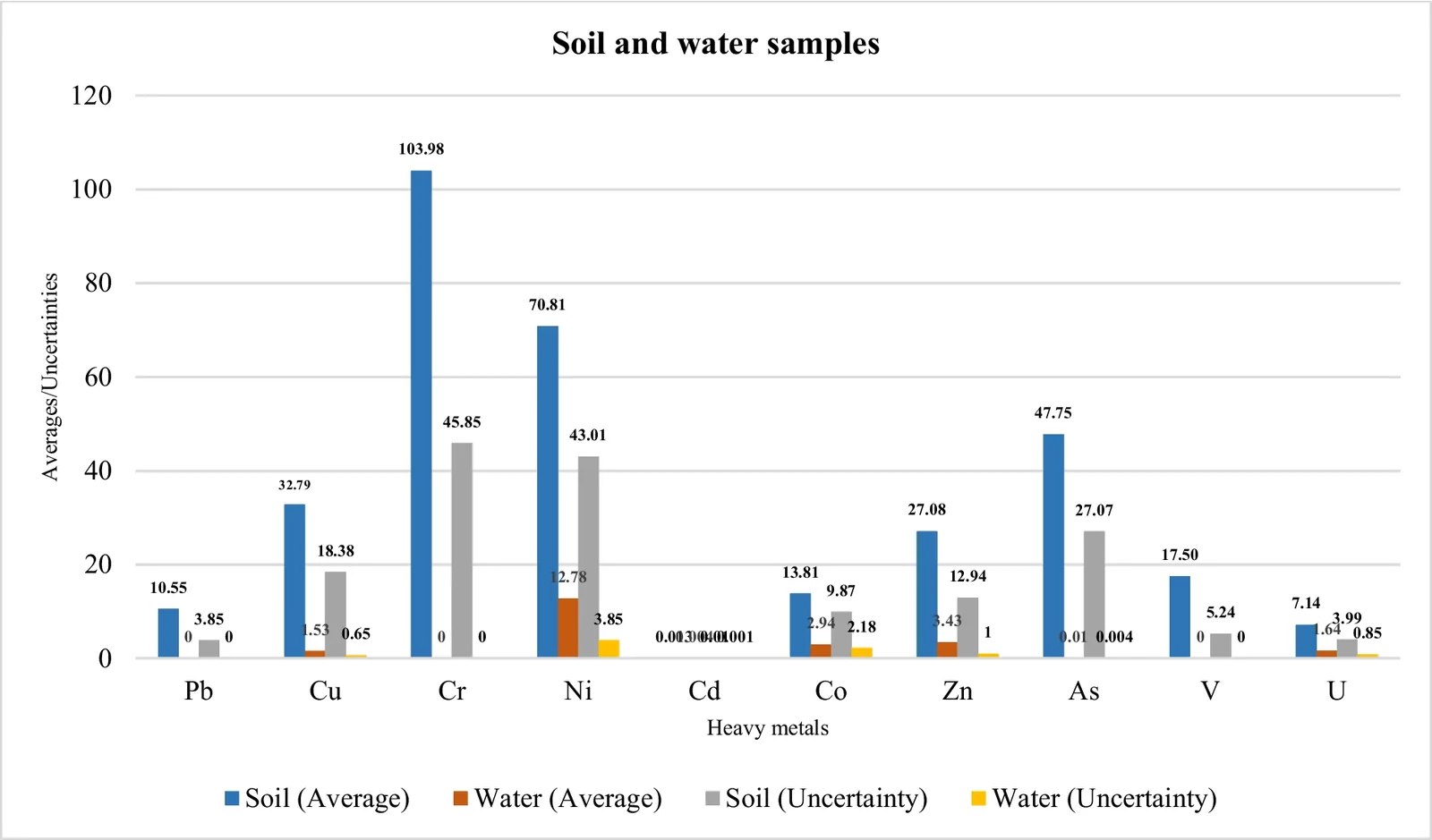
Measured average concentrations and associated uncertainties of heavy metals in soil and surface water samples

Figure 3 demonstrates uncertainty associated with mean concentrations and reveals significant spatial dispersion for several metals in both soil and water samples. High variability was reported for soil Ni (43.01 mg/kg) and Cr (45.85 mg/kg) and for water Co (2.18 mg/L). Conversely, soil V (5.24 mg/kg) and water Pb (below detection limit in all samples) exhibited minimal variability, indicating nonuniform distribution and potential point-sources.

**Fig. 4:**
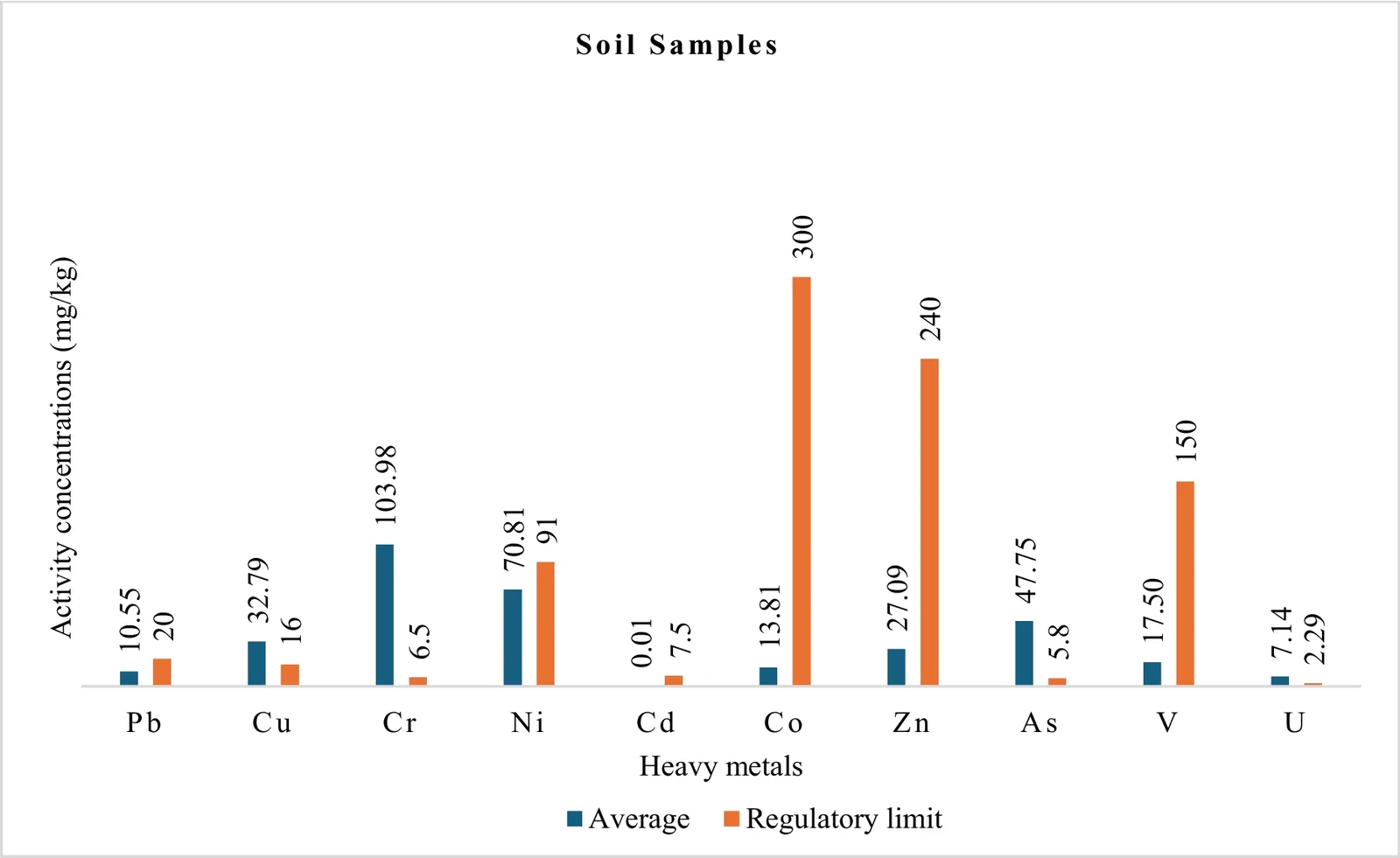
Average concentrations of heavy metals in soil samples compared with the South African National Norms and Standards for Remediation of contaminated land (DFFE, 2014)

As depicted in Fig. 4, the average elemental concentrations of selected heavy metals (As, Pb, Cd, Cr, Cu, Ni, Zn, Co and U) varied across the studied sites. The measured concentrations ranged from 0.01 to 103.98 mg/kg and were compared with the South African acceptable limits provided under the National Norms and Standards for the Remediation of Contaminated Land and Soil Quality (DFFE, 2014).

**Fig. 5:**
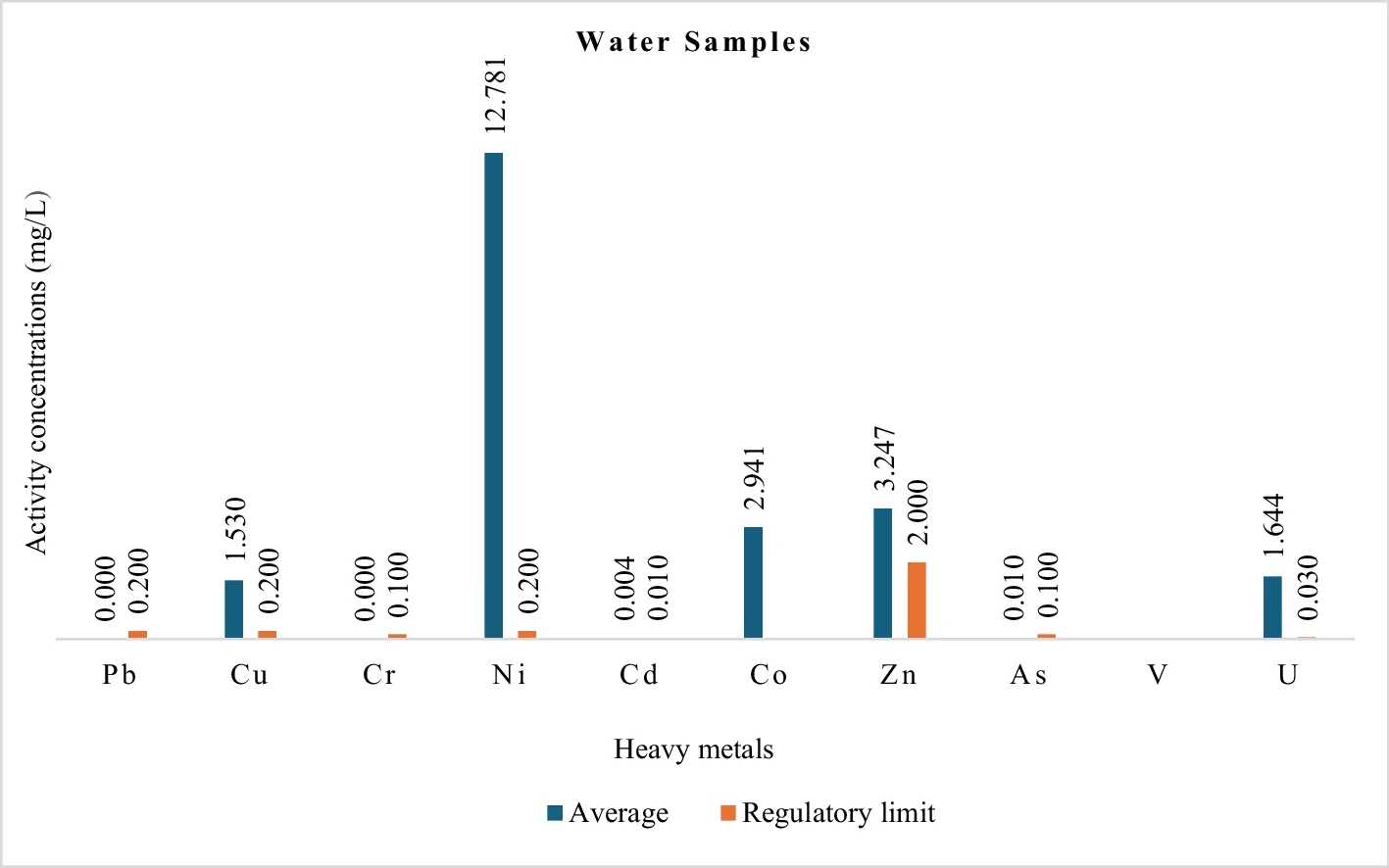
Average concentrations of heavy metals in water samples compared with regulatory limits

As shown in Fig. 5, the average concentrations of selected heavy metals (As, Pb, Cd, Cr, Cu, Ni, Zn, Co and U) in water samples varied across the studied sites, with concentrations ranging from 0 and 12.781 mg/L. The measured concentrations were compared with water quality guidelines for agricultural use and ecological health (WHO, 2017; 2022; DWS, SA. 2021; Drechsel et al., 2023; Odume et al., 2024), as the sampled water bodies are not designated for potable use.

### Radioactivity concentration levels

As shown in Table 3, the activity concentration levels of ^238^U range between 12.78 and 409.73 Bq/kg (mean 160.46 Bq/kg). For ^232^Th, the activity concentrations range from 5.20 to 74.14 Bq/kg with a mean value of 19.65 Bq/kg, whereas ^40^K activity concentration ranges between 13.17 and 704.51 Bq/kg (mean of 376.23 Bq/kg).

**Table 3:** Activity concentrations of NORMs (^238^U, ^232^Th and ^40^K) in soil samples from the Brakpan mine wasteland

Sample ID	^238^U (Bq/kg)	^232^Th (Bq/kg)	^40^K (Bq/kg)
BP1 (control sample)	12.78	9.28	13.17
BKS2	131.88	19.34	378.50
BKS3	64.78	18.22	704.51
BKS4	251.97	21.01	552.78
BKS5	136.07	8.24	373.74
BKS6	47.30	74.14	327.10
BKS7	289.34	19.11	288.50
BKS8	111.07	5.20	164.50
BKS9	264.27	18.69	345.10
BKS10	216.03	20.19	357.80
BKS11	200.08	16.74	517.70
BKS12	78.69	10.24	305.80
BKS13	229.10	12.85	475.00
BKS14	409.73	20.40	696.00
BKS15	172.60	20.95	402.00
BKS16	146.30	16.33	402.90
SD	95.87	13.81	189.68
Median	146.30	18.28	375.62
Min	12.78	5.20	13.17
Max	409.73	74.14	704.51
Mean	**160.46**	**19.65**	**376.23**

All measured activity concentrations exceeded the corresponding MDA values, indicating that the reported results are above detection limits and analytically reliable

Table 4 presents the determined values of radiological indices from soil samples. The absorbed dose rate (*D*) in the control sample (BP1) measured at 12.06 nGy/h, whereas values from mine wasteland sites ranged from 55.29 to 230.64 nGy/h, with a mean of 108.19 nGy/h. The radium equivalent activity (Ra_eq_) was 27.07 Bq/kg in the control sample and increased to a maximum of 492.49 Bq/kg at the most impacted site (BKS12), with an average value of 231.13 Bq/kg across all the *study* sites. The Hₑx ranged between 0.07 and 0.92 with a mean value of 0.62, and *I*_ɣr_ ranged at 0.19 and 3.40 with an average of 1.61.

**Table 4:** Determined values of radiological indices from soil samples

Sample ID	Absorbed dose rate (*D*) nGy/h	Radium equivalent (Ra_eq_) Bq/kg	Hazard indices (*H*ₑ*x*)	Representative level index (*I*_ɣr_)
BP1 (control sample)	12.06	27.07	0.07	0.19
BKS2	88.40	188.69	0.51	1.32
BKS3	70.31	145.09	0.39	1.08
BKS4	152.15	324.59	0.88	2.26
BKS5	83.43	176.64	0.48	1.24
BKS6	80.27	178.51	0.48	1.27
BKS7	157.25	338.88	0.92	2.31
BKS8	61.31	131.17	0.35	0.54
BKS9	147.78	317.58	0.86	2.18
BKS10	126.92	272.45	0.74	1.88
BKS11	124.14	263.88	0.71	1.85
BKS12	55.29	116.89	0.32	0.83
BKS13	133.42	284.05	0.77	1.97
BKS14	230.64	492.49	1.33	3.40
BKS15	109.16	233.51	0.63	1.63
BKS16	94.25	200.67	0.54	1.41
Area SD	50.54	108.31	0.29	0.75
Area median	94.25	200.67	0.54	1.41
Area min	12.06	27.07	0.07	0.19
Area max	230.64	492.49	1.33	3.40
Area mean	108.19	231.13	0.62	1.61

## Discussion

Evaluation of heavy metals and NORMs in relation to mine wasteland contamination in Brakpan confirmed the co-occurrence of significant chemical and radiological contamination. This co-occurrence reflects consistency between chemically measured uranium concentrations (ICP-MS) and elevated radiological activity (gamma-ray spectrometry), although the two methods quantify different properties. ICP-MS analysis confirmed that the Brakpan mine wasteland is contaminated with U, As, Cr and Cu, with concentrations exceeding South African regulatory limits in both soil and water (DFFE, 2014; DWS, SA. 2021). Uranium concentrations averaged 7.14 mg/kg across the study sites, more than three times the South African permissible limit of 2.29 mg/kg in soil, while Arsenic peaked at 47.75 mg/kg, exceeding the national guideline value (5.8 mg/kg) by over eightfold (DFFE, 2014). Cr (103.98 mg/kg) and Cu (32.79 mg/kg) concentrations also exceeded South African soil screening values of 6.5 mg/kg for Cr and 16 mg/kg for Cu for residential soils. These results confirm that the Brakpan mine wasteland represents a major and persistent source of heavy metal pollution, driven largely by long-term tailings weathering and acid AMD processes (Mogashane et al., 2025).

Comparable contamination patterns have been reported in gold mining regions and the Iberian Pyrite Belt, where oxidised sulphide tailings continue to release arsenic and associated metals into surrounding environments (Fereja et al., 2025; Mourinha et al., 2022).

Similar enrichment of As, Cr, Cu, Ni and Zn has been documented in mining-impacted areas of Canada and China, where sulphide oxidation and smelting residues dominate contamination pathways (Anderson et al., 2022; Zhao et al., 2022). These parallels indicate that Brakpan follows a global geochemical pattern where acidic conditions enhance metal mobility and off-site transport. The average U concentration measured in surface water samples (1.64 mg/L) far exceeds the South African water quality guidelines for agricultural use (0.03 mg/L) and ecological health of 0.0003 mg/L (USEPA, 2018; WHO, 2017; 2022; Odume et al., 2024; Du Plessis et al., 2025). Concentrations of Cu, Ni, Co and Zn were also above South African Water Quality guidelines for Aquatic Ecosystems, where chronic effects thresholds for aquatic life are set at 0.003 mg/L (Cu), 0.03 mg/L (Ni), 0.001 mg/L (Co) and 0.005 mg/L (Zn) (USEPA, 2018; Odume et al., 2024). Moderate levels of Cd (0.004 mg/L) and As (0.010 mg/L) were also detected. The presence of these elements at elevated concentrations suggests potential environmental and health implications. Arsenic is widely recognised for its carcinogenic effects, while U presents chemical toxicity, particularly affecting renal function and radiological hazards. Cr may exhibit elevated toxicity depending on its oxidation state, whereas Ni and Co are associated with respiratory and dermal effects under prolonged exposure. Although Cu and Zn are essential trace elements, elevated concentrations may result in adverse ecological effects, particularly in aquatic systems. The elevated U concentrations in surface water are controlled by geochemical conditions, particularly pH and redox potential. Under oxidising acidic conditions typical of AMD, U occurs predominately as the soluble uranyl ion (UO_2_^2+^), enhancing its mobility. Although pH was not measured, the co-occurrence of Cu, Ni, Co and Zn suggests conditions favourable for metal mobilisation. Filtration (0.45 μm) indicates that concentrations represent the dissolved fraction. The metal distribution reflects differences in hydrolysis constants and speciation under acidic conditions. In contrast, uranium concentrations reported from Namibia (Husab), India (Jaduguda) and Germany (Thuringia) generally remain below 1 mg/L (Sharma et al., 2021; Wolkersdorfer et al., 2021; IAEA, 2023; Kumar et al., 2024), highlighting the advanced degree of uranium leaching at Brakpan and the elevated risk of exposure through drinking water, irrigation and food chain transfer.

The co-occurrence of Cu, Ni, Co and Zn in the same water samples mirrors contamination patterns reported from the Laga Dambi gold mining catchment in Ethiopia, the Animas River catchment in the western United States, and the Río Tinto system in the Iberian Pyrite Belt (Martín-Méndez et al., 2023). In these systems, mixed-metal exposure has been linked to long-term health impacts in nearby communities (USEPA, 2025; IAEA, 2023; Alemayehu et al., 2025). These comparisons confirm that the Brakpan mine wasteland is not an isolated case. Instead, it forms part of a broader international pattern of persistent metal pollution associated with abandoned and poorly managed mine residues.

The radiological assessment using absorbed dose rate (*D*), radium equivalent activity (Ra_eq_), external hazard index (*H*ₑ*x*) and the representative gamma index (*I*_γr_) further confirmed elevated exposure risks to surrounding communities. Gamma-ray spectrometry revealed uranium (^23^⁸U) activity concentrations ranging from 12.78 to 409.73 Bq/kg (mean 160.21 Bq/kg), substantially exceeding the global average of 35 Bq/kg. Thorium (^232^Th) ranged from 5.20 to 74.14 Bq/kg (mean 19.65 Bq/kg), while potassium (^4^⁰K) varied between 13.17 and 704.51 Bq/kg (mean 376.23 Bq/kg). While Th and K values were generally within or close to national and global averages. In contrast, uranium levels at Brakpan exceeded those reported from several mining regions, including the Jaduguda tailings complex in India, the Rössing and Husab uranium fields in Namibia and the Colorado Plateau uranium province in the United States (Mathuthu et al., 2021; USGS, 2022; IAEA, 2023; Singh et al., 2025).

The calculated absorbed dose rate D ranged from 12.06 to 230.64 nGy/h, with a mean value of 108.19 nGy/h, which is nearly twice the global average of 59 nGy/h reported by UNSCEAR (2021), indicating enhanced external gamma exposure from the Brakpan tailings. Localised hotspots, particularly BKS12 (230.64 nGy/h), are of critical concern and approach dose rates reported from other Witwatersrand sites such as Kagiso and Soweto, as well as Kitwe, Zambia, where values exceeding 200 nGy/h have been associated with elevated community exposures (Zupunski et al., 2023; Mulwanda & Chilufya, 2024). The presence of these hotspots necessitates immediate and targeted containment strategies that extend beyond simple capping, supporting the use of engineered covers incorporating geomembranes and geotextiles to isolate radioactive materials and prevent further dispersal.

Ra_eq_ values ranged from 27.07 to 492.49 Bq/kg, with a mean of 231.13 Bq/kg indicating that the average value remains below the UNSCEAR recommended threshold of 370 Bq/kg. However, sample BKS12 exceeded this limit, confirming localised radionuclide enrichment. *H*ₑ*x* values indicated unsafe external radiation exposure risk at the same hotspot (*H*ₑ*x* = 1.33), exceeding the recommended safety criterion of *H*ₑ*x* ≤ 1 (NNR, 2021; UNSCEAR, 2021). The *I*_γr_ further identified elevated gamma radiation at BK12 (*I*_γr_ = 3.40), comparable to values reported near Krugersdorp, Carletonville tailings, uranium tailing in Mailuu-Suu (Kyrgyzstan) and Guilan Province (Iran) (Ibrayeva et al., 2024; Kardan et al., 2017; Moshupya et al., 2023), reinforcing the widespread nature of radiological risks associated with gold and uranium mine residues.

Within international context, the levels of heavy metal contamination and radiological risk at Brakpan are comparable to those reported from some of the most severely impacted postmining landscapes globally. These include the Iberian Pyrite Belt in Spain and Portugal, Elliot Lake in Ontario (Canada) and the Laga Dambi gold mining district in Ethiopia (QMENR, 2020; Mourinha et al., 2022; IAEA, 2023; Fereja et al., 2025). However, unlike several of these international sites where structured rehabilitation and long-term containment programmes have been implemented, there are currently no effective mitigation or containment measures in place at Brakpan. This lack of intervention allows the continuous dispersion of both heavy metals and radionuclides into surrounding soils, surface water and groundwater systems (Ilori & Chetty, 2024).

These findings confirm that the Brakpan mine wasteland represents a persistent and unmanaged source of combined chemical and radiological pollution. The associated risks undoubtedly extend beyond the immediate footprint of the site and closely resemble conditions reported from some of the most degraded mining regions worldwide. Without structured containment and rehabilitation, continued exposure through multiple environmental pathways is inevitable, placing surrounding communities at sustained environmental and health risk. The results highlight the need for a remediation framework that is designed for Brakpan’s conditions. Engineered covers such as geomembrane-clay composite barriers would be appropriate for high-risk hotspots where uranium and radiological indices exceed guideline limits (USEPA, 2018; Bussière et al., 2022; CER, 2025). These structures can reduce erosion, limit radon release and prevent further mobilisation of radium and uranium decay products by more than 80%, as demonstrated in uranium tailings in Namibia and Canada (Jones et al., 2020). For larger areas with moderate contamination, phytoremediation offers a practical option that promotes soil stability, contaminant uptake and ecological recovery while creating employment opportunities through community-based restoration.

The elevated concentrations of residual uranium and gold suggest that remining could serve both environmental and economic purposes. Removing contaminated tailings at their source would reduce the long-term hazard and generate resources that can offset rehabilitation costs. This aligns with emerging approaches in South Africa where remining has become part of integrated closure strategies (Engineering News, 2024; Harmony Gold, 2024).

Overall, the findings illustrate that Brakpan (Kwa-Thema) as a mine-impacted area requires remedial measures and can serve as a model for development of sustainable management of mine wastelands in SA. A combined strategy such as hotspot containment, site-wide phytoremediation and selective remining offers the most balanced path to reducing community exposure, improving land usability and supporting broader development goals. These interventions directly align with the Sustainable Development Goals that promote health (SDG-3), clean water (SDG-6) and sustainable human settlements (SDG-11).

## Study limitations

This study was based on a single sampling campaign and therefore does not account for seasonal variations in contaminant concentrations. Groundwater was not assessed, and geochemical parameters such as pH and redox potential were not measured, limiting detailed evaluation of contamination mobility. In addition, the radiological assessment focused on external gamma exposure and did not include radon measurements or detailed human health risk assessment. These limitations should be considered when interpreting the findings.

## Conclusion

This study assessed heavy metals and NORMs associated with historical mine wastelands in Brakpan (Kwa-Thema), South Africa, using ICP-MS and gamma-ray spectrometry. The results showed elevated activity concentrations of U, As, Cr and Cu in soil and water samples, exceeding South African and global reference levels at several points. Radiological assessment further revealed elevated uranium activity concentrations, absorbed dose rates and hazard indices, with hotspot BKS12 exceeding recommended safety limits. The co-occurrence of chemical and radiological contamination indicates that the Brakpan mine wasteland remains a source of environmental pollution with potential implications for surrounding ecosystems and communities. The findings are consistent with the long-term environmental impacts of unmanaged historical mine residues and highlight the importance of integrated monitoring and sustainable management of mine wastelands in South Africa.

## Recommendations of the study

High-risk zones such as BKS12 need engineered containment to limit radiation exposure and prevent further contaminant dispersion. Wider areas with moderate contamination can be stabilised through phytoremediation, which supports soil recovery and community-based ecological restoration. The presence of recoverable uranium and gold also opens the possibility of selective remining as a way to remove contaminated materials while offsetting rehabilitation costs. Brakpan can serve as a benchmark for understanding impacts of mine wastelands and motivation for sustainable mine wasteland management across the Witwatersrand Basin.

## Data Availability

No datasets were generated or analysed during the current study.
